# PhenomeScape: a cytoscape app to identify differentially regulated sub-networks using known disease associations

**DOI:** 10.1093/bioinformatics/btw545

**Published:** 2016-08-24

**Authors:** Jamie Soul, Sara L. Dunn, Tim E. Hardingham, Ray P. Boot-Handford, Jean-Marc Schwartz

**Affiliations:** Faculty of Biology, Medicine and Health, University of Manchester, Manchester M13 9PT, UK

## Abstract

**Summary:** PhenomeScape is a Cytoscape app which provides easy access to the PhenomeExpress algorithm to interpret gene expression data. PhenomeExpress integrates protein interaction networks with known phenotype to gene associations to find active sub-networks enriched in differentially expressed genes. It also incorporates cross-species phenotypes and associations to include results from animal models of disease. With expression data imported into PhenomeScape, the user can quickly generate and visualise interactive sub-networks. PhenomeScape thus enables researchers to use prior knowledge of a disease to identify differentially regulated sub-networks and to generate an overview of altered biologically processes specific to that disease.

**Availability and Implementation:** Freely available for download at https://github.com/soulj/PhenomeScape

**Contact:** jamie.soul@postgrad.manchester.ac.uk or jean-marc.schwartz@manchester.ac.uk

## 1 Introduction

The interpretation of gene expression data to gain insights into molecular mechanisms of disease after differentially expression analysis is challenging. Looking at groups of genes using enrichment or network based methods adds power to the analysis and aids interpretation. Gene set enrichment approaches are limited to predefined sets of genes and ignore the known interaction information. Finding *de novo* sub-networks/pathways from a protein–protein interaction (PPI) network and the expression data itself is an alternative approach employed by many tools (Ideker *et al.*, 2002). For many diseases, there are genes, which are known through human Mendelian disease and animal models, where perturbation gives rise to observed phenotypes present in the disease under study. Projects such as the International Knockout Mouse Consortium are adding much new information as they systematically identify gene to phenotype associations (Bradley *et al.*, 2012). This prior knowledge is valuable, since when combined with the gene expression data it indicates which genes and pathways/regions of the interactome are of importance in the disease.

We recently described an algorithm named PhenomeExpress for analysis of disease related expression data that makes use of known cross-species phenotype to gene associations to generate sub-networks of differentially expressed genes that are linked to the observed phenotypes (Soul *et al.*, 2015). We here present PhenomeScape, a Cytoscape application allowing a user to quickly run the described PhenomeExpress algorithm on gene expression data within the Cytoscape GUI environment (Shannon *et al.,* 2003).

## 2 Methods

### 2.1 Network sources

The UberPheno network ontology was used to produce a phenotype-phenotype similarity network using Resnik semantic similarity, with a cut-off of 3 to keep strong interactions between phenotypes (Köhler *et al.*, 2013). Gene to cross-species phenotype associations were downloaded from UberPheno. Example high confidence mouse and human networks from HumanConsensusPathDB and StringDB are provided for convenience and can be loaded though the app menu (Kamburov *et al.*, 2009; von Mering *et al.*, 2003). All IDs were mapped to official gene symbols using Biomart (Smedley *et al.*, 2015).

### 2.2 PhenomeScape input

**Fig. 1.**
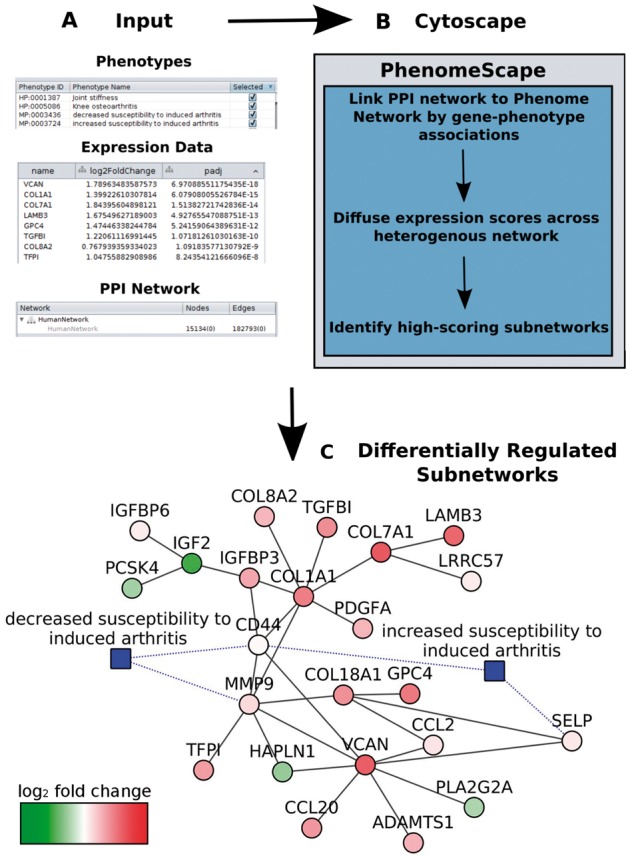
Schematic representation of the PhenomeScape app. **(A)** Example input data. **(B)** Pipeline for differentially regulated sub-network identification. **(C)** Example output of sub-network derived from the input PPI. Protein nodes are coloured by fold change (red increase, green decrease). Direct interactions between chosen phenotypes that describe the disease under study to their associated genes are indicated in blue

For input PhenomeScape requires a PPI network, either input using standard Cytoscape import methods or a provided one (Fig. 1A). Analysed expression data with gene symbols, fold change and *P*-values are imported into Cytoscape and matched with the network by the Gene Symbol. No thresholding by fold change or *P*-value is required as significance is determined at a sub-network level by calculation of empirical *P*-values through random sampling of the background network. Network parameters are set to sensible defaults, but can be altered to increase the size and number of the resulting sub-networks for further exploration of the data. The min sub-network size prevents creation of very small sub-networks while the sub-network size parameter is a constant introduced in the PhenomeExpress algorithm that influences the maximum size of the sub-networks. The *P*-value threshold allows filtering of the sub-networks by their statistical significance. Finally, phenotypes describing the disease under study are selected from a table with a filter function.

### 2.3 Sub-network identification

The PhenomeExpress algorithm has previously been described in detail. Briefly, the input PPI is linked to a provided phenotype-phenotype network via phenotype-gene associations (Fig. 1B). The expression data and the chosen phenotypes are used with a diffusion approach to calculate activity scores of proteins in the PPI. Subsequently, highly scoring groups of interacting proteins are identified as sub-networks.

### 2.4 PhenomeScape output

PhenomeScape creates all identified sub-networks for visualisation, coloured to indicate fold change for rapid identification of the most differentially expressed genes (Fig. 1C). Direct associations between the chosen phenotypes and the genes are shown allowing the user to view which genes have evidence of being previously linked to the disease phenotypes. The results panel shows the empirical *P*-value of the sub-networks and to the top enriched GO term to give an indication of the function of the *de novo* sub-networks.

## 3 Use case

Previously analysed gene expression data comparing human damaged and intact osteoarthritic knee cartilage was downloaded from ArrayExpress (E-MTAB-4304). This data was analysed as previously described to produce a table of all expressed genes and their corresponding fold changes and *P*-values (Dunn *et al.*, 2016). The default human network derived from human ConsensusPathDB was loaded from the PhenomeScape app menu. Using the standard table import tools the expression data spreadsheet was loaded and added to the network in Cytoscape by using the gene symbol as a key. The resulting human network has nodes labelled with expression data where the gene is expressed. Phenotypes relevant to the disease osteoarthritis were selected using the text box to filter the phenotype selection table. Increased and decreased susceptibility to arthritis, joint stiffness and knee osteoarthritis phenotypes were chosen based on review of the available phenotype terms.

Using default network parameters, PhenomeScape identified 21 statistically significantly sub-networks including a sub-network with the top GO term enrichment of organisation of the extracellular matrix, shown in Figure 1C. This is consistent with the known perturbation of the extracellular matrix during osteoarthritis. Four phenotypes to associated gene links are shown in this sub-network, several genes of which are strongly differentially expressed demonstrating the utility of incorporating the prior disease knowledge.

## 4 Conclusion

PhenomeScape allows users to rapidly explore analysed gene expression data. Compared to existing tools such as ReactomeFIViz, PhenomeScape is unique in allowing integration of the prior phenotypic knowledge of disease genes into the expression analysis. No coding experience is needed to run PhenomeScape making it suitable for bench scientists who have gene expression data available. The resulting sub-networks produced in Cytoscape allow the user to extend the analysis with additional apps for regulatory analysis. PhenomeScape provides new insights in active processes revealed by expression data in the context of the prior knowledge of the disease phenotypes.
